# The Influence of verbalization on the pattern of cortical activation during mental arithmetic

**DOI:** 10.1186/1744-9081-8-13

**Published:** 2012-03-12

**Authors:** Sabrina Zarnhofer, Verena Braunstein, Franz Ebner, Karl Koschutnig, Christa Neuper, Gernot Reishofer, Anja Ischebeck

**Affiliations:** 1 Cognitive Psychology and Neuroscience, Department of Psychology, University of Graz, Universitaetsplatz 2/III, 8010 Graz, Austria; 2 Department of Radiology, Medical University of Graz, Auenbruggerplatz 9, 8036 Graz, Austria; 3 Department of Knowledge Discovery, University of Technology of Graz, Krenngasse 37/IV, 8010 Graz, Austria; 4 Department of Psychology, University of Graz, Universitaetsplatz 2, 8010 Graz, Austria

**Keywords:** fMRI, Cognitive styles, Number processing, Gyrus supramarginalis, Heschl's gyrus, Rolandic operculum

## Abstract

**Background:**

The aim of the present functional magnetic resonance imaging (fMRI) study at 3 T was to investigate the influence of the verbal-visual cognitive style on cerebral activation patterns during mental arithmetic. In the domain of arithmetic, a visual style might for example mean to visualize numbers and (intermediate) results, and a verbal style might mean, that numbers and (intermediate) results are verbally repeated. In this study, we investigated, first, whether verbalizers show activations in areas for language processing, and whether visualizers show activations in areas for visual processing during mental arithmetic. Some researchers have proposed that the left and right intraparietal sulcus (IPS), and the left angular gyrus (AG), two areas involved in number processing, show some domain or modality specificity. That is, verbal for the left AG, and visual for the left and right IPS. We investigated, second, whether the activation in these areas implied in number processing depended on an individual's cognitive style.

**Methods:**

42 young healthy adults participated in the fMRI study. The study comprised two functional sessions. In the first session, subtraction and multiplication problems were presented in an event-related design, and in the second functional session, multiplications were presented in two formats, as Arabic numerals and as written number words, in an event-related design. The individual's habitual use of visualization and verbalization during mental arithmetic was assessed by a short self-report assessment.

**Results:**

We observed in both functional sessions that the use of verbalization predicts activation in brain areas associated with language (supramarginal gyrus) and auditory processing (Heschl's gyrus, Rolandic operculum). However, we found no modulation of activation in the left AG as a function of verbalization.

**Conclusions:**

Our results confirm that strong verbalizers use mental speech as a form of mental imagination more strongly than weak verbalizers. Moreover, our results suggest that the left AG has no specific affinity to the verbal domain and subserves number processing in a modality-general way.

## Background

As individuals differ with regard to their potentials, habits and preferences, they differ in their approach to complex problems, and the degree to which they employ different cognitive domains in solving a complex problem. It is unclear, however, how individual preferences in problem solving are reflected in the brain. We will focus here on the distinction between visual and verbal cognitive styles because this distinction has repeatedly been used to categorize solution alternatives, ability profiles and processing differences in mathematics and arithmetic processing [1-3].

### The visual and verbal cognitive style

A cognitive style is assumed to be a relatively stable characteristic that describes how an individual processes information, that is, how an individual thinks, perceives, and remembers [4]. Verbalizers report to repeat information during thinking verbally, whereas visualizers claim to represent information during thinking pictorially or schematically. In the domain of arithmetic, a visual style might mean to visualize numbers and (intermediate) results while calculating or to move mentally along the mental number line. A visual style might also mean for example imagining a mass that increases or decreases in magnitude, or some collection of dots that grows or shrinks in number. In the domain of arithmetic, a verbal style might mean that numbers and (intermediate) results are verbally repeated during calculation.

The visual and verbal cognitive style was first conceptualized as a bipolar construct with the preference to visual or verbal ways of information processing portrayed as two contrasting poles. Although this categorization of individuals into visualizers and verbalizers is intuitively convincing, the independence of brain areas subserving verbal and visual processing makes it implausible to assume that good visualizers have to be bad verbalizers and vice versa, as implied in the one-dimensional model of cognitive styles. Accordingly, Blazhenkova and Kozhevnikov [5] suggested independence of visualization and verbalization during information processing. A given individual might as well be visualizing as verbalizing during information processing but might show a preference for visualization or verbalization.

To our knowledge, however, there are only two fMRI studies so far that investigated the impact of visual and verbal cognitive styles on cognitive performance and brain activation. An fMRI study by Burbaud et al. [6] using an arithmetic task found brain activation differences between visualizers and verbalizers in areas involved in verbal and visual processing, but not in brain areas associated with number processing. It should be noted that the authors assessed cognitive styles using a one-dimensional 5-point rating scale (1...purely verbal/5...purely visual). The authors assumed thus the one-dimensional model with two contrasting poles of cognitive styles as the basis for their study. As described above, the one-dimensional model was replaced by a multi-dimensional model, and the results of the study of Burbaud et al. should hence be interpreted with great care. A recently published fMRI study by Kraemer et al. [7] observed that individuals tend to mentally convert information that is presented in a not preferred mode to the preferred mode of processing. In this study, the cognitive style of an individual was assessed with the Verbalizer-Visualizer-Questionnaire [8]. The authors observed that during reading, visualizers showed activation in the right gyrus fusiformis, an area implicated in visual processing, whereas during picture presentation, verbalizers showed activation in the left gyrus supramarginalis, an area implicated in verbal processing. The findings indicate that individuals with a visual cognitive style could have a tendency to convert linguistically presented information into a visual mental representation. Similarly, individuals with a verbal cognitive style could have a tendency to convert pictorially presented information into a verbal mental representation. These studies suggest that general differences between verbalizers and visualizers exist with regard to brain activation patterns, but it is yet unclear whether these individual differences also affect brain areas specifically involved in arithmetic number processing.

### The neural basis of number processing and arithmetic

In recent years, research with imaging and other neuroscientific methods has greatly advanced our understanding of the neural basis of number processing. Humans as well as animals were found to have a basic concept of number and order, and they can roughly estimate [9]. Results from fMRI studies [10,11] as well as single unit recordings in monkeys [12] have shown that the anterior part of the intraparietal sulcus (IPS), bilaterally, is crucially involved in the processing of numbers. Specifically, some researchers have proposed that the IPS hosts an amodal representation of quantity, which has been referred to as the 'mental number line' [13,14]. In arithmetic, the IPS plays a role for example in solving subtraction problems. Whereas other researchers have proposed that the IPS shows some modality specificity as the IPS also plays a role in visual attention [15-17], and in visual-spatial short-term memory [18,19]. Dehaene and colleagues [20], however, proposed that the IPS is not domain-specific but rather amodal and hence not necessarily a visual processing area. Another brain area important for arithmetic problem solving is the left angular gyrus (AG), which is assumed to support the long term memory retrieval for arithmetic fact knowledge [20]. Arithmetic fact knowledge is required, for example, in the skilled solving of multiplication problems by retrieving the result from verbal long-term memory, namely, from the multiplication tables learned in childhood. This has lead researchers to believe that the AG might show some affinity to the verbal domain [20]. Some recent findings, however, raise some doubts about the assumption that the left AG mediates verbal fact retrieval during multiplication. No activation above control condition, arithmetic tasks performed with Roman numerals, was found during mental arithmetic tasks performed with Arabic numerals in either AG region [21]. In another fMRI study, multiplication and subtraction problems differed significantly in right, but not left, IPS and AG activity [22]. A meta-analyses showed that the ability to process numbers and perform calculations relies on a large number of brain regions [23]. In addition to the brain areas of the triple-code model [20], activation during number and calculation tasks were also observed in the cingulate gyri, the insula, and the cerebellum. Furthermore, activation in dorsolateral and frontopolar areas of the prefrontal cortices was modulated by task difficulty [23].

### The present study

If an individual's cognitive style reflects a general preference for a stimulus modality, we hypothesized, first, that strong verbalizers show higher activation in brain areas associated with language (supramarginal gyrus, Broca's area) and auditory (Heschl's gyrus, Rolandic operculum) processing than weak verbalizers, and that strong visualizers show higher activation in brain areas associated with visual processing (fusiform gyrus, visual cortex) than weak visualizers while solving arithmetic problems.

If the brain areas involved in number processing are domain-specific (IPS) or modality-specific (AG) as proposed in the triple code model [20], that is, that the left AG shows an affinity to the verbal domain, and that the left and right IPS shows an affinity to the visual domain, the activation of these areas should show a dependency on an individual's cognitive style. We hypothesized, second, that strong verbalizers show higher activation within the left AG than weak verbalizers, and that strong visualizers show higher activation within the IPS, bilaterally, than weak visualizers.

The present study comprised two functional sessions in an event-related design. In the first functional session, subtraction and multiplication problems were presented, and in the second functional session, multiplications were presented in two formats, with Arabic numerals or with written number words. The individual's habitual use of visualization and verbalization during mental arithmetic was assessed with a short self-report measure. Additionally, to assess a possible correlation between cognitive style and intelligence, the participants completed an intelligence test in a separate session.

In the first functional session, multiplication and subtraction problems were presented. When these different kinds of problem types are compared as a function of cognitive style, we expected, first, higher activation within the IPS, bilaterally, while solving subtraction problems. This activation might be higher in strong visualizers compared to weak visualizers. We expected, second, higher activation within the left AG while solving multiplication problems. This activation might be higher in strong verbalizers compared to weak verbalizers.

In the second functional session, multiplication problems were presented in two formats. When these different types of format are compared as a function of cognitive style, we expected, first, higher activation within brain areas associated with language processing while solving problems that are presented with Arabic numerals for strong verbalizers compared to weak verbalizers, because strong verbalizers tend to convert visually presented information into a verbal mental representation. We expected, second, higher activation within areas that are involved in visual processing while solving problems that are presented with written number words for strong visualizers compared to weak visualizers, because strong visualizers tend to convert verbally presented information into a visual mental representation.

The present fMRI study aims at expanding our present general knowledge of brain function in the field of arithmetic problem solving to better understand and manage the consequences of interindividual differences.

## Methods

### Participants

42 healthy young adults (21 female) participated in the fMRI study. All were right-handed, all had normal or corrected to normal vision, and no history of neurological or psychiatric illness. All participants were university students. The participants had an average age of 23 years (*S.D*. = 3.22). All participants were tested during daytime. The study was approved by the ethics committee of the Medical University Graz.

### Self-report measure

We designed a short self-report measure to assess the use of verbalization and visualization in mental arithmetic in a preliminary study. From an initial number of ten items five were excluded because they failed quality criteria (e.g., difficulty, selectivity). From the remaining five items, two items addressed the use of visualization in mental arithmetic (e.g., In my mind, I see the numbers and (intermediate) results as written on paper.), and three items addressed the use of verbalization in mental arithmetic (e.g., I solve a problem easier if I repeat the problem and the (intermediate) results in my mind.). The participants of the present study had to evaluate themselves for all items on a five point rating scale. The ticked values for the two visual items were added and divided by two. The ticked values for the verbal items were added and divided by three. As a consequence the self-reported use of verbalization and visualization was comparable.

### Intelligence test

The intelligence test was conducted to assess the relationship between cognitive style and intelligence to be sure that the assessed use of visualization and verbalization during mental arithmetic of an individual does not depend on intelligence. We used a standardized intelligence test (Intelligence Structure Test in German language: I-S-T 2000 R) [24]. The battery contains a basic module measuring verbal, numerical, and figural intelligence. Each content area of intelligence is assessed through three subtests.

### Stimuli

For the first functional session, multiplication and subtraction problems were designed. 15 multiplications were chosen from the multiplication table. All were one-digit times one-digit problems with two-digit solutions. The one-digit operands were 3, 4, 6, 7, 8, and 9. Problems with other operands (1, 2 or 5) and ties (two equal operands, e.g. 4 × 4) were not presented [25]. The order of the factors of the 15 multiplication problems was changed (e.g., 3 × 4 and 4 × 3) yielding 30 multiplication problems in total. The 30 multiplication problems were presented twice during the first functional scan. Distractors for the multiplication problems were operand-related (i.e. solutions of related problems; e.g., the distractors of the problem 3 × 4 would be 8 (2 × 4), 16 (4 × 4), 9 (3 × 3), and 15 (3 × 5)). Subtractions were chosen such that their mean problem size was equal to the mean problem size of the multiplication problems. The minuend, the subtrahend (13, 14, 16, 17, 18 or 19) and the difference were two-digit numbers (e.g., 36-19). The 15 selected subtraction problems were each presented four times during the first functional session. To prevent the use of shortcut strategies, distractors for the subtraction problems were chosen such that the differences to the correct solution (± 1 or ± 10) were balanced over all problems and participants. Multiplication and subtraction problems were intermixed and presented in random order. The first functional session consisted of 120 trials, 60 multiplication and 60 subtraction problems. The mean problem size (i.e. solution) of those multiplication and subtraction problems was equal.

For the second functional session, the same 30 multiplication problems as in the first functional session were used. Each problem was presented twice in different formats, namely, either as Arabic numerals (e.g., 3 × 4) or as written number words (e.g., three times four). The number word problems were presented in German. The distractors were operand-related. The distractor and the solution were presented in the same format as the problem. A total of 60 trials were presented in random order.

### Procedure

Before entering the scanner, the participant had to evaluate all items of the self-report measure to assess his use of verbalization and visualization in mental arithmetic.

**Figure 1:**
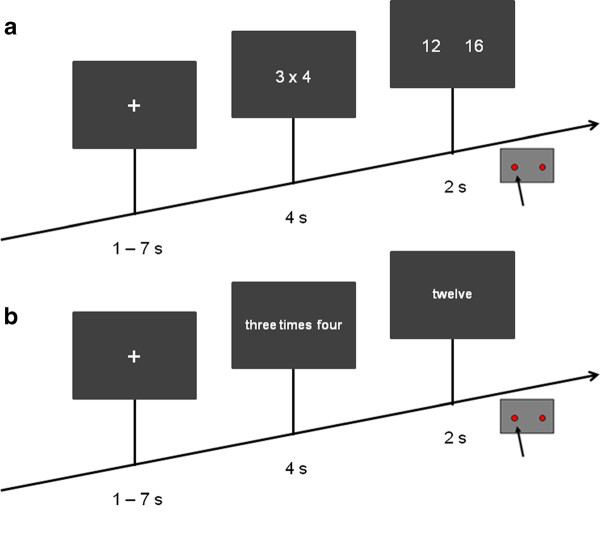
**Schematic of the task**. **A**) First functional session: Subtraction and multiplication problems were presented in randomized order (120 trials). Participants had to choose the alternative that corresponds the solution. Response times were measured from the onset of the presentation of the alternatives. **B**) Second functional session: 60 multiplication problems were presented as Arabic numerals or as written number words in German language. Participants had to indicate if the solution is correct or not. Response times were measured from the onset of the presentation of the solution.

During the first functional session, the participant had to solve 60 multiplication and 60 subtraction problems presented in random order. Each trial started with the presentation of a fixation cross for 1 to 7 seconds (s; average presentation time of 4 s), followed by the presentation of the problem for 4 s. Then the two alternatives - the solution and one of the distractors - were presented for 2 s, giving a total trial duration of 9 to 13 s. The scheme of the task is shown in Figure 1A. Response times were measured from the onset of the presentation of the two alternatives. The participant had to indicate on which side of the screen the correct response was presented by pressing the corresponding button with his right hand. All stimuli were presented in white letters against a grey background. The first functional session lasted approximately 20 minutes.

During the second functional session the participant had to solve 30 multiplication problems in two presentation formats, namely with Arabic numerals or with written number words. Each trial started with the presentation of a fixation cross for 1 to 7 s (average presentation time of 4 s), followed by the presentation of the problem for 4 s. The result was shown for 2 s, giving a total trial duration of 9 to 13 s. The scheme of the task is shown in Figure 1B. Response times were measured from the onset of the presentation of the result that could be correct or incorrect. The participants were instructed to use their right index finger to press on one of the two buttons depending on whether the answer is correct or not. All stimuli were presented in white letters against a grey background. The second functional session lasted approximately ten minutes.

Stimulus presentation and response recording were programmed with the software Presentation (Neurobehavioral Systems).

On a different day the participants took the intelligence test (Intelligence Structure Test in German language, I-S-T 2000 R) [24]. This test took approximately 90 minutes to complete.

### fMRI data acquisition

Imaging was preformed with a 3 T Siemens Magnetom Tim Trio scanner (Siemens Medical Solutions, Erlangen, Germany) and a 32-channel head coil. For the anatomical images, an isotropic MPRAGE sequence was used (TR = 1900 ms, TE = 2.19 ms, TI = 900 ms, flip angel = 9° inplane acqisition matrix = 256 × 256, FoV = 256 (saggital view), 176 partitions, slice thickness 1 mm). For the functional images, a T2*-weighted EPI-sequence was employed (TR/TE = 2000 ms/24 ms, matrix = 64 × 64, FoV = 192, 31 axial slices, inplane resolution: 3 × 3 mm, slice thickness 3 mm and 0.90 mm gap, acquired descendingly and parallel to the AC-PC line) sensitive to brain oxygen-level dependent (BOLD) contrast. 31 axial contiguous slices parallel to the bicommissural plane were acquired, covering the whole brain. Participants wore ear plugs as protection against the scanner noise.

### fMRI data analysis

Data analyses and pre-processing were performed with the SPM software (SPM 8, Wellcome Department of Cognitive Neurology, London, U.K.). The first two functional images of each participant were discarded to allow for magnetic saturation. The remaining functional images were motion-corrected, unwarped, and corrected for slice acquisition time. The functional images were then normalized to correspond more closely to the MNI anatomical template. Images were finally smoothed with a Gaussian kernel of 8 mm FWHM.

Statistical analyses were performed on the basis of the general linear model implemented in SPM8. A model with two conditions for each functional scan was analyzed (first functional session: multiplication/subtraction; second functional session: Arabic format/word format). The experiment was analyzed on the basis of single events (event-related). The trial onsets of the single events were calculated from the logfiles saved after presenting the two experimental conditions with the software Presentation (Neurobehavioral Systems) for each participant separately. The delta-function of the trial onsets for each condition was convolved with the canonical form of the hemodynamic response function and its first and second derivative. A high-pass filter of 1/200 Hz and an autocorrelation model (AR(1)) were employed, but no low-pass filter and no global normalization. For the statistical group analyses, one sample t-tests were calculated to realize a random effects analysis. Significant activation clusters were determined using a height threshold of *p *< .001 uncorrected, with family-wise error (FWE) correction for multiple comparisons at *p *< .05 and an uncorrected voxelwise *p *< .001 level.

### Region of interest analyses

For our ROI analysis, we focused on brain areas implicated in language, auditory, visual, and number processing. The ROIs selected for analysis were the right and left fusiform gyrus (BA 37; visual processing), supramarginal gyrus (BA 40; language processing), Heschl's gyrus (BA 41; auditory processing), Rolandic operculum (BA 43; auditory processing), visual cortex (BA 17/18/19; visual processing), and intraparietal sulcus (number processing) as well as the left Broca's area (BA 44/45; language processing), and the left angular gyrus (BA 39; number processing). The ROIs were defined on the basis of the AAL atlas [26]. For the ROI analysis, the Marsbar toolbox was used (M. Brett, http://marsbar.sourceforge.net). Effect sizes were averaged over all voxels of the ROI on the individual participant level, for each condition against baseline. Next, we tested for correlations between these effect sizes and values of the self-reported use of verbalization and visualization.

### Behavioural data analysis

For the behavioural data analysis, only the data of those participants who were entered into the functional data analysis were used. For the first functional session, five participants (three female) had to be excluded from the analysis because of excessive motion. The response times data of one subject had to be excluded because of a technical malfunction. For the first functional session, the behavioural data were analyzed for the remaining 37 participants (18 female; age: *M *= 23.11, *S.D*. = 3.23). For the second functional session, six participants (four female) had to be excluded from the analysis because of excessive motion. For the second functional session, the behavioural data were analyzed for the remaining 36 participants (17 female; age: *M *= 23.03, *S.D*. = 3.09). For the analysis of the response times, response times below 200 ms were considered outliers. In total 386 data points for the first functional session (from 4440 data points in total), and 201 data points for the second functional session (from 2160 data points in total) were removed from the analysis. Error rates were arcsin √p transformed to achieve approximate variance equality [27]. Response times and the arcsin √p transformed error rates were entered into two separate paired t-tests with the factors OPERATION (multiplication, subtraction) for the first functional session, and FORMAT (Arabic, word) for the second functional session.

## Results

With regard to intelligence, we observed no significant correlation between the self-reported use of verbalization or visualization in mental arithmetic and verbal, numerical or figural intelligence. This indicates that an self-reported habitual use of visualization and verbalization during mental arithmetic does not depend on intelligence. Statistics for the correlations between the cognitive style and intelligence are presented in Table 1.

**Table 1:** Correlations between verbalization/visualization and intelligence

	Verbal intelligence	Numerical intelligence	Figural intelligence
Visualization	-.066 (*p *= .678)	-.090 (*p *= .572)	-.125 (*p *= .429)

Verbalization	.114 (*p *= .472)	.223 (*p *= .156)	.141 (*p *= .374)

The self-reported use of verbalization (*M *= 3.46, *S.D*. = 1.07) and visualization (*M *= 3.91, *S.D*. = .93) is correlated negatively (*r *= .-456, *p *= .002; *n *= 42).

### Response times and error rates

In the first functional session, multiplication problems were solved faster (766 ms vs. 904 ms) and with fewer errors (4.57% vs. 9.08%) than subtraction problems (response times: *t*35 = -6.46, *p *< 0.001; error rates: *t*36 = -4.71, *p *< 0.001).

In the second functional session, multiplication problems presented with Arabic numerals were solved faster (863 ms vs. 1126 ms) than multiplication problems presented with written number words (response times: *t*36 = 1.0 *p *< 0.001). No difference was observed for the error rates (error rates: 4.25% vs. 4.56%, n.s.).

### Whole brain analyses

**Figure 2:**
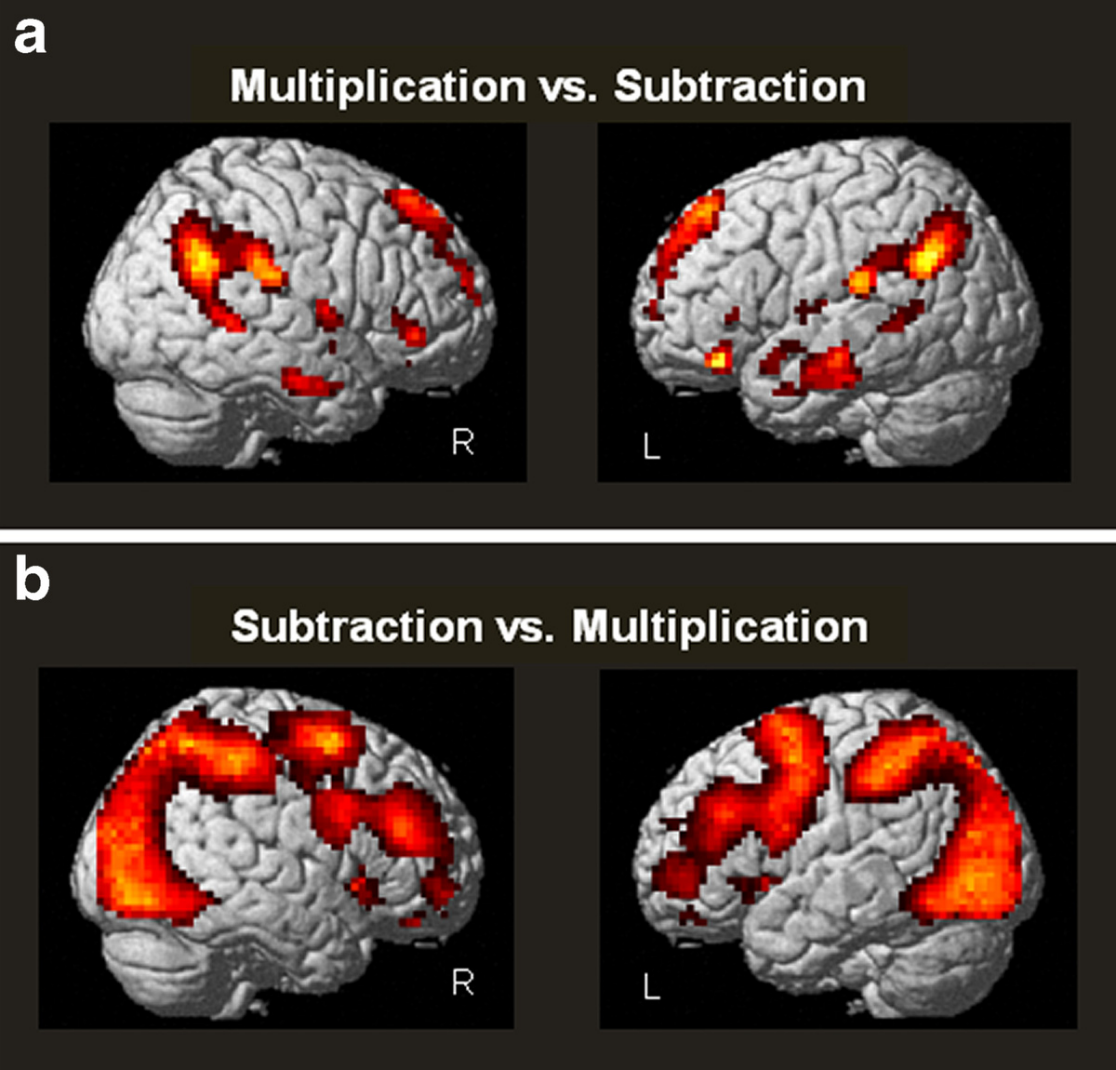
**Whole Brain Analysis: First functional session**. **A**) One-sample *t*-test of the contrast multiplications versus subtractions (*p *< 0.001). **B**) One-sample *t*-test of the contrast subtrations versus multiplications (*p *< 0.001).

In the first functional session, we compared two operations, multiplication and subtraction problems. Compared to multiplication problems, subtraction problems activated a range of areas in the frontal and parietal lobe, including the IPS, as well as the basal ganglia. In the reverse contrast, more activation was observed within the left and right AG, and in frontal brain areas (see Table 2 and Figure 2).

**Table 2:** First functional session: Group analysis of the contrasts subtraction versus multiplication and multiplication versus subtraction

Side	Sub > Mult	x	y	z	k	Z	Side	Mult > Sub	x	y	z	k	Z
right	Parietal_Sup	15	-64	52	1465*	Inf	left	Angular	-54	-64	34	369	6.25

left	Parietal_Sup	-12	-67	52	866*	Inf	right	Angular	57	-58	34	502	6.05

right	Caudate	21	14	4	247*	Inf	left	Frontal_Sup	-12	41	49	364	5.57

left	Frontal_Mid	-24	-1	55	114*	7.30	left	Frontal_Inf_Orb	-42	32	-17	85	5.04

left	Putamen	-18	14	1	160*	7.27	left	Temporal_Inf	-57	-22	-26	142	4.92

right	Frontal_Sup	27	2	52	84*	7.10	right	Cingulum	9	-49	31	194	4.88

left	Occipital_Inf	-42	-79	-2	204*	7.00	right	Frontal_Sup	15	35	55	199	4.67

left	Thalamus	-12	-19	10	19*	6.49	left	Insula	-39	-13	7	158	4.36

left	Supp_Motor_Area	3	14	52	48*	6.49							

left	Precentral	-45	5	31	12*	6.40							

right	Lingual	24	-55	-2	2*	6.23							

right	Thalamus	15	-19	10	1*	6.10							

left	Occipital_Mid	-24	-97	-2	3*	6.07							

(Abbreviations: k = cluster size, Z = *Z*-value; activation significant at *p *< 0.001 uncorrected, *p *< 0.05 FWE corrected on cluster level)

Coordinates are reported as given by SPM8 (MNI space) and correspond only approximately to Talairach and Tournoux space (Talairach and Tournoux, 1988, Brett et al., 2001). The label denotes the location of the maximum. Abbrevations: Parietal_Sup - superior parietal lobe, Caudate - caudate nucleus, Frontal_Mid - middle frontal gyrus, Frontal_Sup - superior frontal gyrus, Occipital_Inf - inferior occipital gyrus, Supp_Motor_Area - supplementary motor area, Precentral - precentral gyrus, Lingual - lingual gyrus, Occipital_Mid - middle occipital gyrus, Angular - angular gyrus, Frontal_Inf_Orb - inferior orbital frontal gyrus, Temporal_Inf - inferior temporal gyrus, Cingulum - cingulate gyrus, * this activation is part of a bigger cluster

**Figure 3:**
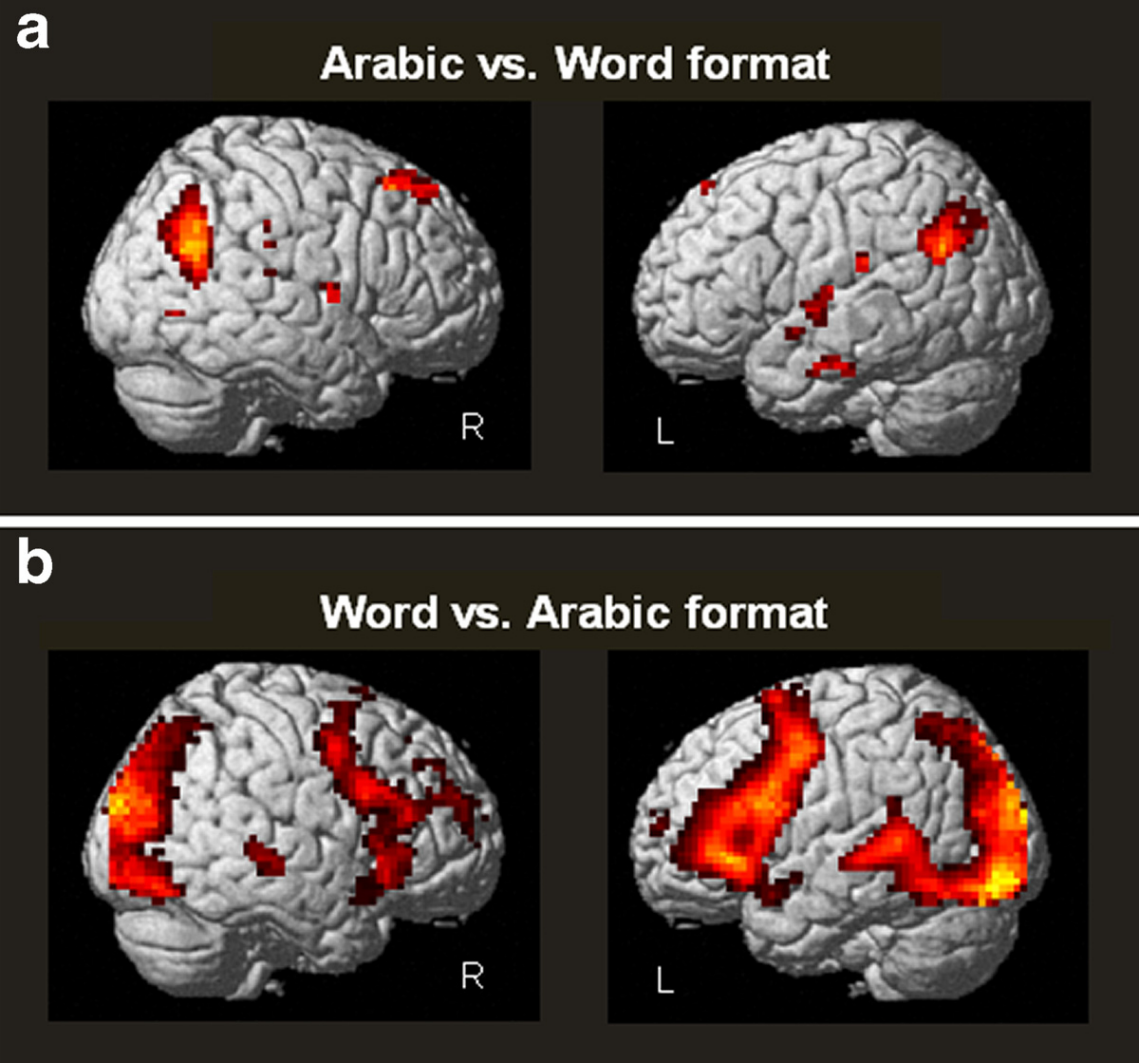
**Whole Brain Analysis: Second functional session**. **A**) One-sample *t*-test of the contrast Arabic versus word format (*p *< 0.001). **B**) One-sample *t*-test of the contrast word versus Arabic format (*p *< 0.001).

In the second functional session, we compared multiplication problems presented with Arabic numerals with the same problems presented with written number words. Compared to problems presented with Arabic numerals, problems presented with written number words activated more strongly inferior frontal brain areas including Broca's area, temporal brain areas including the left superior temporal gyrus, parietal brain areas including the left and right AG, IPS, the fusiform gyrus, and supramarginal gyrus, and occipital brain areas. In the reverse contrast the left and right AG, the left posterior cingulum, the right superior frontal gyrus, and the left insula were activated (see Table 3 and Figure 3).

**Table 3:** Second functional session: Group analysis of the contrasts word versus Arabic format and Arabic versus word format

Side	Word > Ara	x	y	z	k	Z	Side	Ara > Word	x	y	z	k	Z
left	Calcarine	9	-79	1	7028	Inf	right	Angular	48	-58	28	209	5.06

left	Frontal_Inf_Oper	-39	8	25	2597	Inf	right	Cingulum_Post	9	-49	28	192	5.05

right	Frontal_Inf_Oper	45	11	28	693	5.09	right	Frontal_Sup	33	26	55	84	5.03

right	Temporal_Mid	48	-34	1	94	5.07	left	Angular	-51	-64	31	141	4.04

right	Frontal_Inf_Orb	39	23	-8	294	4.99	left	Insula	-33	16	4	101	3.98

right	Thalamus	12	-4	-5	77	4.22							

(Abbreviations: k = cluster size, Z = *Z*-value; activation significant at *p *< 0.001 uncorrected, *p *< 0.05 FWE corrected on cluster level)

Coordinates are reported as given by SPM8 (MNI space) and correspond only approximately to Talairach and Tournoux space (Talairach and Tournoux, 1988, Brett et al., 2001). The label denotes the location of the maximum. Abbrevations: Calcarine - calcarine gyrus, Frontal_Inf_Oper - inferior frontal gyrus, Temporal_Mid - middle temporal gyrus, Frontal_Inf_Orb - inferior orbital frontal gyrus, Angular - angular gyrus, Cingulum_Post - posterior cingulum, Frontal_Sup - superior frontal gyrus

No significant correlations were observed in the whole brain analysis between brain activation and the values of the self-reported use of visualization or verbalization.

### ROI analyses

Correlations were calculated between averaged effect sizes within each ROI and the values of the self-reported use of verbalization as well as visualization in mental arithmetic. We used a Bonferroni correction for the number of ROIs for each modality. Thus, the critical *p *value for the verbal cognitive style hypotheses is .006 (.05/8; 8 independent ROIs: left angular gyrus, left supramarginal gyrus, right supramarginal gyrus, left Heschl's gyrus, right Heschl's gyrus, left Rolandic Operculum, right Rolandic Operculum, and left Broca's area), and the critical *p *value for the visual cognitive style hypotheses is .005 (.05/10; 10 independent ROIs: left inferior visual cortex, left middle visual cortex, left superior visual cortex, right inferior visual cortex, right middle visual cortex, right superior visual cortex, left fusiform gyrus, right fusiform gyrus, left IPS, and right IPS).

In the first functional session, correlations were calculated between averaged effect sizes within each ROI for multiplication and subtraction problems and the values of the self-reported use of verbalization as well as visualization in mental arithmetic. Significant correlations were observed only for the subtraction problems, and the scores of the self-reported use of verbalization. We found significantly positive correlations between scores on the self-reported use of verbalization and one area implicated in verbal processing, the right and left supramarginal gyrus (right: *r *= .364, *p *= .027; left: *r *= .462, *p *= .004, Bonferroni-corrected), and one area implicated in auditory processing, namely the left Rolandic operculum (*r *= .361, *p *= .028). These results suggest that the higher the self reported tendency to verbalize, the higher the activation in an area implicated in language processing, namely the left and right supramarginal gyrus, and an area implicated in auditory processing, namely the left Rolandic operculum, while solving subtraction problems. It should be noted, however, that only the correlation with the left supramarginal gyrus passes the Bonferroni correction.

**Figure 4:**
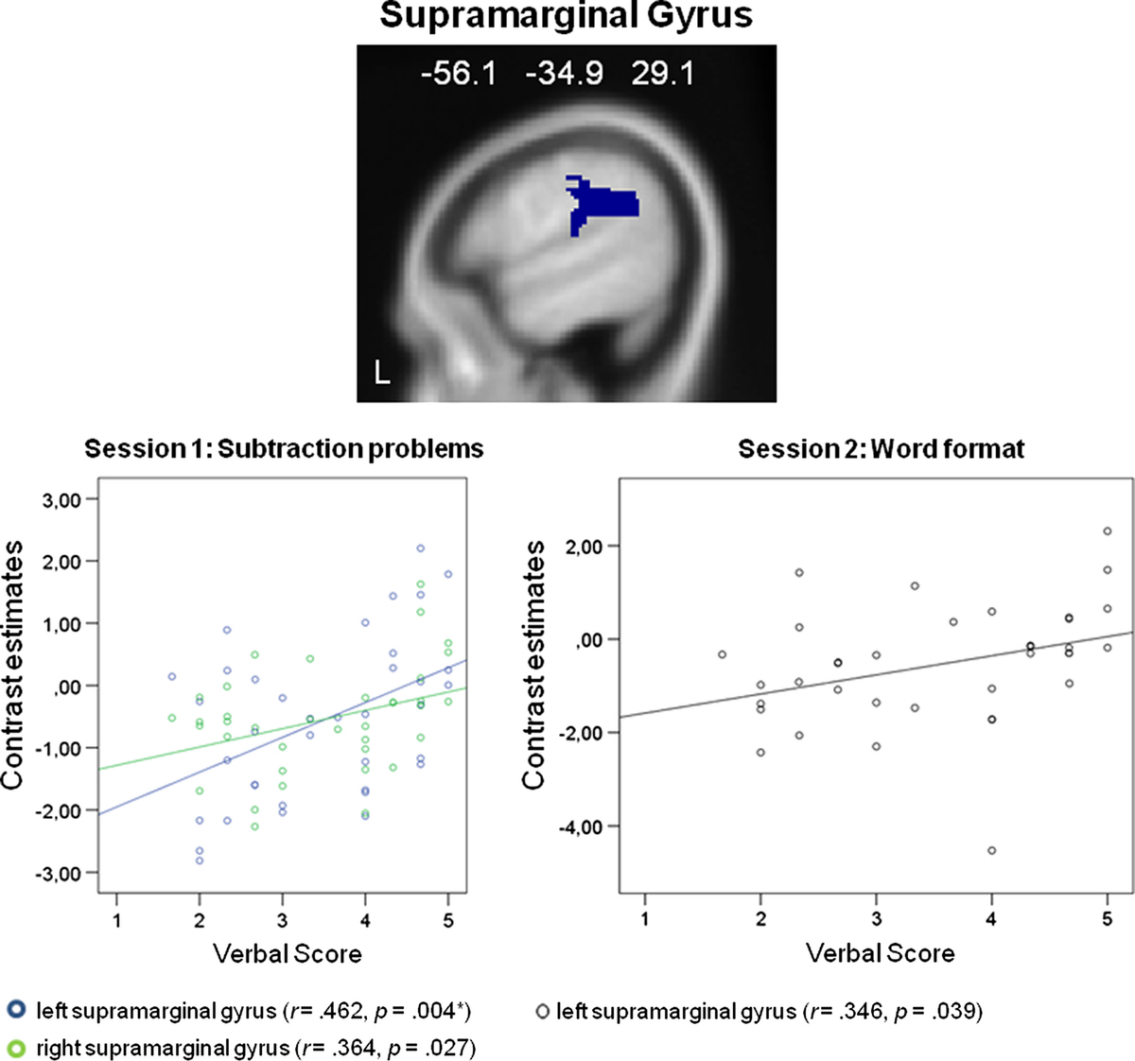
**ROI analysis results: Supramarginal Gyrus**. Significant correlation between peak activations in the supramaginal gyrus and the self-reported use of verbalization: In the first functional session significant correlations appeared for the subtraction problems and the right and left [* correlation passes the Bonferroni correction (critical *p *value is .006)] supramarginal gyrus. In the second functional session a significant correlation appeared for multiplications presented with written number words and the left supramarginal gyrus.

**Figure 5:**
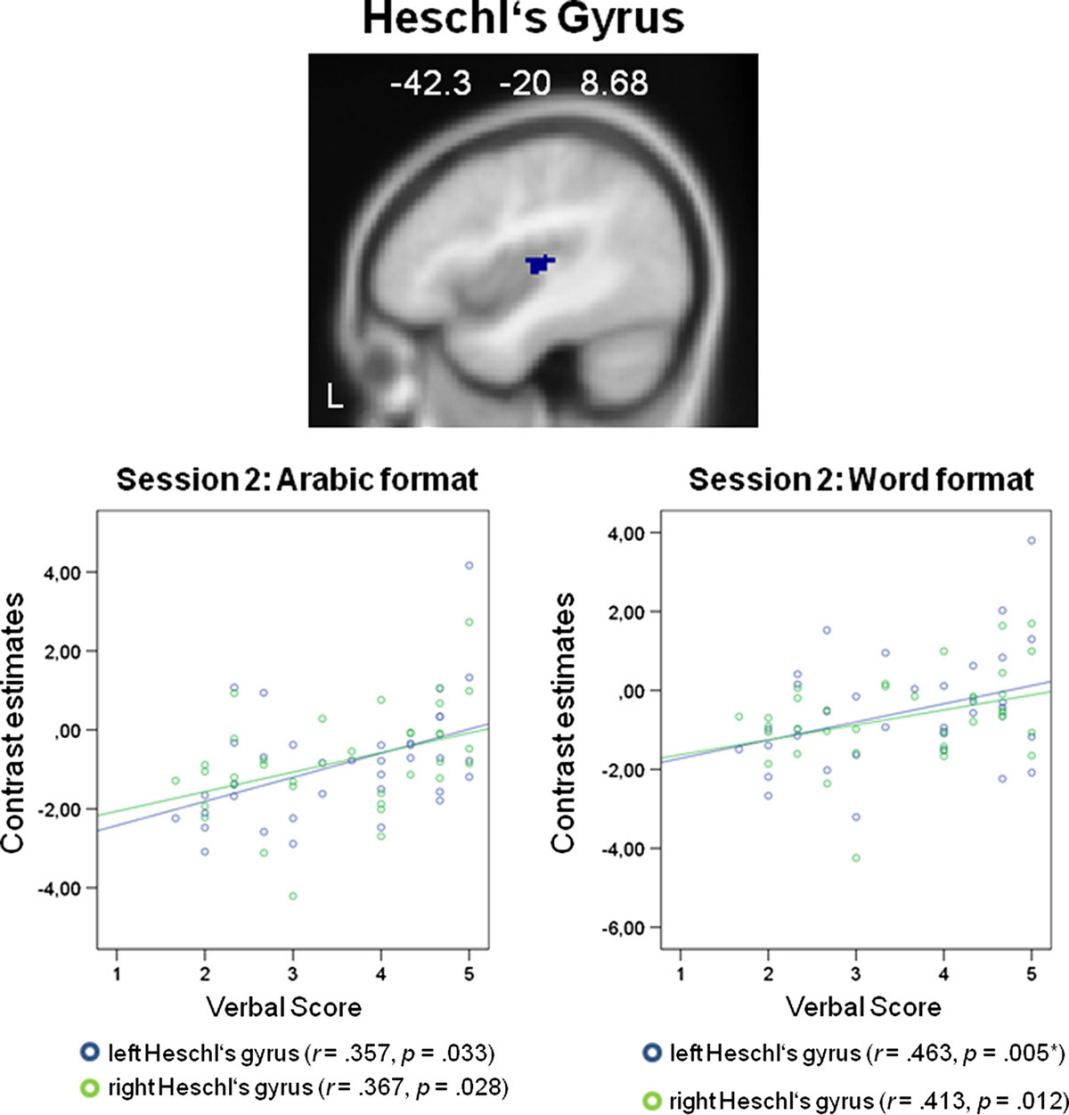
**ROI analysis results: Heschl's Gyrus**. Significant correlations between peak activations in the Heschl's gyrus and the self-reported use of verbalization: In the second functional session significant correlations appeared for multiplications presented with written number words and the right and left [* correlation passes the Bonferroni correction (critical *p *value is .006)] Heschl's Gyrus, and for multiplications presented with Arabic numerals and the right and left Heschl's Gyrus.

**Figure 6:**
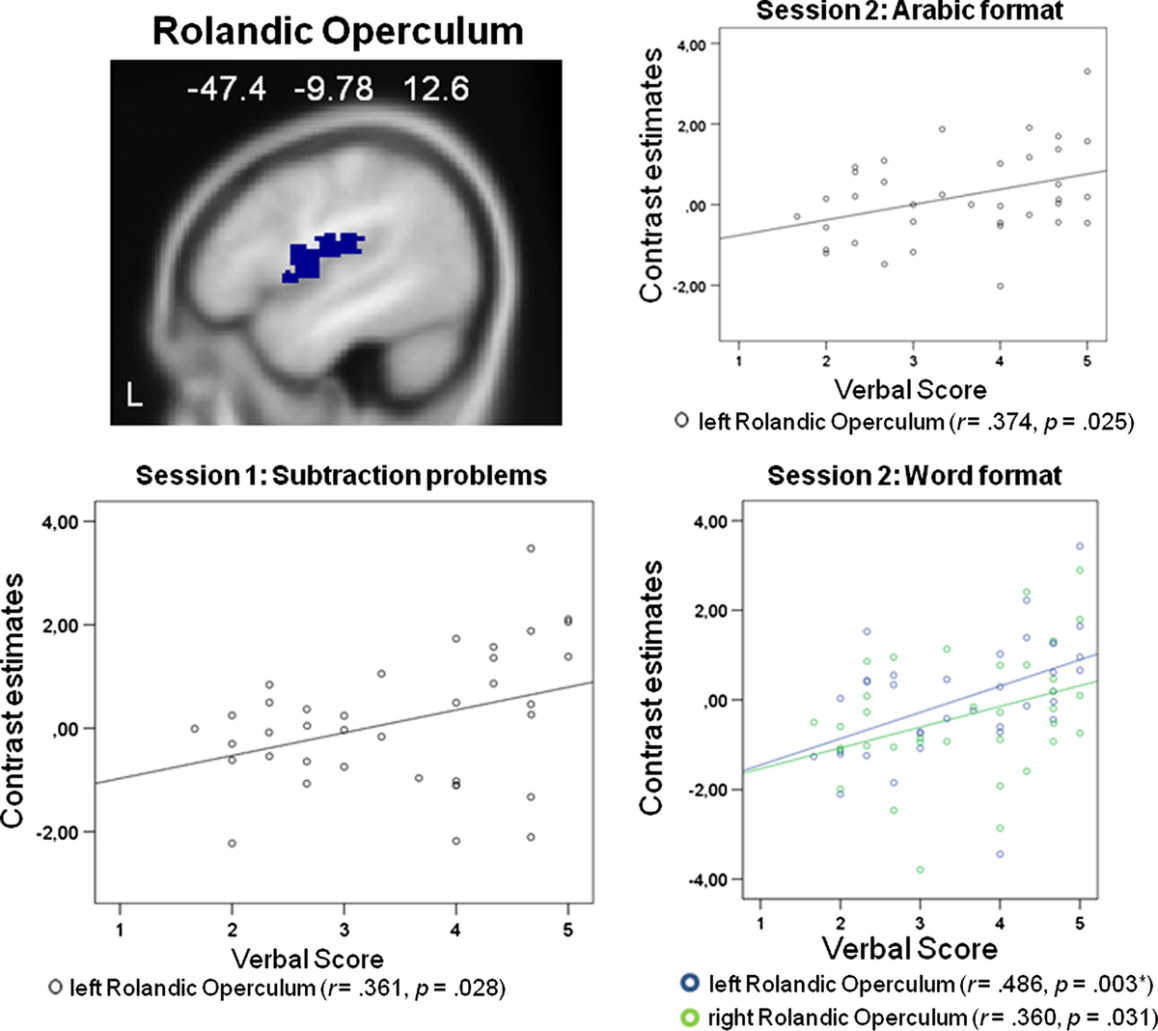
**ROI analysis results: Rolandic Operculum**. Significant correlations between peak activations in the Rolandic operculum and the self-reported use of verbalization: In the first functional session a significant correlation appeared for the subtraction problems and the left Rolandic Operculum. In the second functional session significant correlations appeared for multiplications presented with written number words and the right and left [* correlation passes the Bonferroni correction (critical *p *value is .006)] Rolandic Operculum, and for multiplications presented with Arabic numerals and the left Rolandic Operculum.

In the second functional session, correlations were calculated between averaged effect sizes within each ROI for problems presented with Arabic numerals and problems presented with written number words and the values of the self-reported use of verbalization as well as visualization in mental arithmetic. Significant correlations were observed for both presentation formats. For problems presented with Arabic numerals we found significantly positive correlations between the self-reported use of verbalization and activation in ROIs implicated in auditory processing, the right and left Heschl's gyrus (right: *r *= .367, *p *= .028; left: *r *= .357, *p *= .033), and the left Rolandic Operculum (*r *= .374, *p *= .025). For problems presented with written number words we found significant positive correlations between the self-reported use of verbalization and areas implicated in language and auditory processing, the left supramarginal gyrus (*r *= .346, *p *= .039), the right and left Heschl's gyrus (right: *r *= .413, *p *= .012; left: *r *= .463, *p *= .005, Bonferroni-corrected), and the right and left Rolandic operculum (right: *r *= .360, *p *= .031; left: *r *= .486, *p *= .003, Bonferroni-corrected). We observed no significant correlations for the self-reported use of visualization. These results suggest that the higher the self reported tendency to verbalize while solving multiplication problems the higher the activation in areas implicated in verbal and auditory processing, namely in the left supramarginal gyrus, the right and left Heschl's gyrus, and the right and left Rolandic operculum. It should be noted, that only the correlations between problems presented with written number words and the left Heschl's gyrus as well as the left Rolandic Operculum passed the Bonferroni correction. Figure 4 shows the significant results for the supramarginal gyrus, Figure 5 for the Heschl's gyrus, and Figure 6 for the Rolandic operculum.

## Discussion

The aim of the present fMRI study was to investigate the influence of the verbal-visual cognitive style on cerebral activation patterns during mental calculation. The habitual use of visualization and verbalization during mental arithmetic of each of the 42 right-handed participants was assessed with a short self-report measure. In the first functional session, subtraction and multiplication problems were presented, and in the second functional session, multiplications were presented in two formats, either as Arabic numerals or as written number words.

With regard to verbalization, we found that the higher the self reported tendency to verbalize during mental arithmetic the higher the activation in brain areas related to language or auditory processing, namely, within the right and left supramarginal gyrus, the right and left Rolandic operculum, and the right and left Heschl's gyrus. The supramarginal gyrus is a region in the inferior parietal lobe, and has been found to be involved in phonological processing [28], reading both in regards to meaning [29] and phonology [30], word production [31], and grammar learning [32]. The Rolandic operculum has been found to be a somatosensory region [33,34], involved in auditory processing, activated by listening to the sound of one's own voice [35], and the processing of prosody [36,37]. The left Rolandic operculum is assumed to be involved in syntactic encoding during speaking [38], and phonological rehearsal [39]. The right Rolandic operculum has been associated with the processing of sentence intonation [40], and of slow prosodic modulations [41]. The activation of the right Rolandic operculum increased with the degree an individual relied on verbalization during mental arithmetic when the problems were presented with written number words. This result suggests that verbalizers imagine hearing the sound of their voices mentally while reading numbers presented with number words, confirming their own subjective self-report. The right and left Heschl's gyrus is found in the area of the primary auditory cortex in the superior temporal gyrus of the human brain, the first cortical structure to process incoming auditory information [42]. Naming numbers has been shown to be dependent on linguistic properties. For example, naming latencies for two-digit numbers increase with syllable length [43,44]. The authors suggested that digits are translated into a verbal code before being processed [43,44].

It is unclear, why we found significant correlations only for the verbalizer dimension but not for the visualizer dimension. One reason might be that participants are not very good at self-reporting their habits in information processing. We think it would be a major contribution for further studies in this field to design a more valid questionnaire about cognitive styles in mental arithmetic. A limitation of the present study might be that the self-reported use of visualization in mental arithmetic was limited to the visualization of numbers and (intermediate) results while calculating. Other types of visualizations, such as to move mentally along the number line, imagining a mass that increases or decrease in magnitude, or some collection of dots that grows or shrinks in number and others were not included. Another reason may be that the statistical spread of the self-reported use was greater for the verbalizer dimension than for the visualizer dimension (Var_(score verb = 1.15) _> Var_(score vis = .87) _).

**Figure 7:**
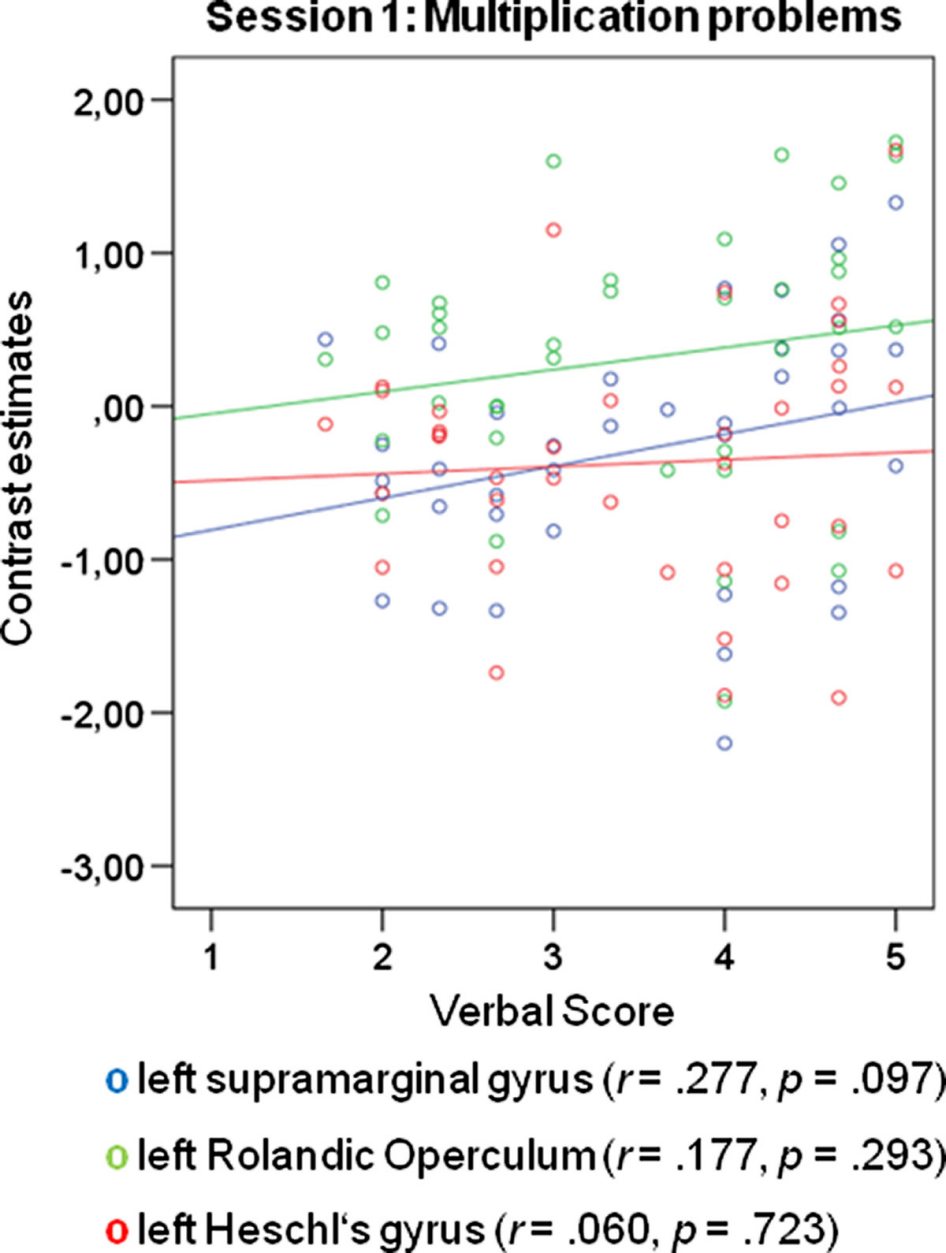
**ROI analysis results: Multiplication problems in the first functional session**. Correlations between peak activations in the left supramaginal gyrus, the left Rolandic Operculum, and the left Heschl's gyrus and the self-reported use of verbalization: In the first functional session no significant correlations appeared for the multiplication problems and the left supramarginal gyrus, the left Rolandic Operculum, and the left Heschl's gyrus.

It is surprising that the correlations for the first functional session between individual differences in verbalization and brain activation during calculation were only found for the subtraction but not for the multiplication problems (see Figure 7). It has frequently been argued that multiplication relies more on verbal processing than subtraction, which is assumed to rely more on visuo-spatial processing [2,45]. Therefore, we would have expected verbalizers to show greater language-related activation for multiplications. One possibility why we did not find significant activation patterns for the multiplication problems in the first functional session could be that the multiplication problems used here were very easy to solve. They were part of the multiplication tables and the results could be retrieved from long-term memory. The behavioral results showed that the presented subtraction problems were more difficult than the presented multiplication problems. It is possible that differences with regard to verbalization as cognitive style emerge only for more difficult arithmetic problems that have to be solved in several processing steps leading to intermediate results that have to be kept in memory.

With regard to a possible domain-specificity (IPS) or modality-specificity (AG) of brain areas involved in number processing we did not observe any modulation. Although we found significant brain activation as a function of verbalization in areas related to sound and language processing, we observed no modulation of activation in the left AG as a function of the self-reported tendency to use verbalization while performing mental arithmetic. The left AG is assumed to support the long term memory retrieval for arithmetic fact knowledge [20]. Arithmetic fact knowledge is required, for example, in the skilled solving of multiplication problems by retrieving the result from the verbal long-term memory, namely, from the multiplication tables learned in childhood. The left AG shows stronger activation for solving arithmetic problems for which participants report fact retrieval whereas the application of procedural strategies is accompanied by widespread activation in a fronto-parietal network [46]. These findings link the left AG to arithmetic fact retrieval. In the present study, the activation of the left AG was not modulated by verbalization, and it could be concluded that the left AG shows no specific affinity to the verbal domain and subserves number processing in a modality-general way. This interpretation corresponds to some recent findings [21,22] which also raise some doubts about the assumption that the left AG mediates verbal fact retrieval during multiplication.

In the first functional session, we found that subtraction problems activated the right and left IPS more strongly than multiplication problems, whereas multiplication problems activated the left and right angular gyrus more strongly than subtraction problems. This finding corresponds well to results reported in previous studies [47,48] as well as to the model of Dehaene and collegues [20]. It should be noted, however, that multiplication problems were solved faster and more accurately than subtraction problems, although problem sizes were identical. It can therefore not be excluded that some of the observed activation differences are also due to a difference in task difficulty.

In the second functional session, we found that multiplications presented with written number words activated areas implicated in visual processing more strongly than multiplications presented with Arabic numerals. It is highly likely that the greater activation in visual areas for multiplications presented with written number words is due to the larger number of characters. The multiplications in written number words were presented using three words with several letters, whereas the multiplications presented with Arabic numerals consisted of two Arabic numerals and a multiplication sign. Other studies also reported format effects in arithmetic. It has been suggested that numbers presented in different surface-formats have differential access to number representations [49]. Format differences were also observed in an EEG-study, with more negative event-related potentials for written number words than for Arabic numerals and auditorily presented number words [50]. The modality-dependent access to numerical information may be a consequence of modality-dependent access to the number representation in parietal cortex. A transcranical magnetic stimulation experiment showed a dissociation between digits and number words in the right parietal lobe, whereas the left parietal lobe showed a double dissociation between the different numerical formats [51]. Typically, problems presented with number words take longer to solve than problems presented with Arabic numerals [52,53]. In our study, multiplications presented with Arabic numerals were also solved faster than multiplication problems presented with written number words.

The visual-verbal cognitive style is assumed to be a relatively stable characteristic although it might depend on the task [5]. An individual might, for example, prefer visualization for solving arithmetic problems and verbalization for memorizing a poem. It is therefore preferable to assess the visual-verbal cognitive style specifically for the tested task domain. We tried to assess the visual-verbal cognitive style during mental arithmetic with a short self-report measure. Our results indicate that people who say they verbalize more show more activity in brain areas related to language and auditory processing. It is unclear, however, in how far these results are specific to mental arithmetic.

A cognitive style is assumed to be a relatively stable characteristic that describes an individual's way to process information [4]. Consequently a questionnaire that assesses the habitual use of visualization and verbalization has to measure an independent construct and not personality or intelligence [54,55]. Previous studies already showed independence of the visual-verbal cognitive style from intelligence and personality [56,57]. Regarding intelligence, we found no significant correlations between the verbal or visual cognitive style and verbal, numerical or figural intelligence. This indicates that the correlations between cognitive style and brain activation are not due to differences in intelligence.

The aim and results of the present study might be understood by some researchers as advocating a view that different learning styles have real consequences for the brain and that education should be adapted accordingly. It is important to distinguish between cognitive styles and learning styles. Cognitive styles are assumed to be an individual's way to process information, whereas learning styles are concerned with the learning environment [58]. Some studies showed correlations between cognitive styles and some environmental conditions, especially the preferred learning mode. There is a tendency for visualizers to use pictures and for verbalizers to prefer writing as working mode or learning mode [59,60]. In our study, the self-reported cognitive style influenced activity in brain areas directly related to the preferred modality of information processing.

## Conclusions

In the present study, we observed that the self-reported habitual use of verbalization in mental arithmetic correlates positively with activation in brain areas implicated in verbal and auditory processing. However, the absence of a similar modulation of activation within areas specifically involved in number processing could be taken to indicate that these areas are less modality-specific (AG) or domain-specific (IPS) than currently proposed. Our results indicate that different cognitive styles do not lead to a difference in arithmetic processing in the brains of skilled adults. It is possible, however, that cognitive styles play a role in the acquisition of mathematic skills. Further studies could show that identifying and catering to a student's preferred cognitive style in education leads to a more efficient acquisition of arithmetic skills.

## Competing interests

All authors declare not to have any conflict of interest including any financial, personal or other relationships with other people or organizations that could inappropriately influence, or be perceived to influence, their work.

## Authors' contributions

The presented work was carried out in collaboration between all authors. All authors read and approved the final manuscript. SZ designed the experiment, collected and analyzed the data, interpreted the results, and wrote the paper. VB helped at collecting the data and gave writing assistance. AI and CN made substantial contributions to the design, the acquisition of data, and the analysis and interpretation of data. FE, KK and GR provided technical help, and gave writing assistance at the method section.
